# Glycemic Control and Implant Stability in Patients with Type II Diabetes: Narrative Review

**DOI:** 10.3390/healthcare13050449

**Published:** 2025-02-20

**Authors:** Saverio Cosola, Andrea Butera, Abenezer Hailu Zergaw, Jaibin George, Ugo Covani, Augusto Arrighi, Paolo Toti, Andrea Scribante, Giovanni Battista Menchini-Fabris

**Affiliations:** 1 Department of Stomatology, Tuscan Stomatologic Institute, Foundation for Dental Clinic, Research and Continuing Education, 55041 Camaiore, Italy; saverio.cosola@unicamillus.org (S.C.); abjeho@gmail.com (A.H.Z.); drjaibin@gmail.com (J.G.); covani@covani.it (U.C.); capello.totipaolo@gmail.com (P.T.);; 2 Department of Dentistry, Unicamillus—Saint Camillus International University of Health and Medical Sciences, 00100 Rome, Italy; 3 Unit of Dental Hygiene, Section of Dentistry, Department of Clinical, Surgical, Diagnostic and Pediatric Sciences, University of Pavia, 27100 Pavia, Italy; andrea.scribante@unipv.it; 4 Unit of Orthodontics and Pediatric Dentistry, Section of Dentistry, Department of Clinical, Surgical, Diagnostic and Pediatric Sciences, University of Pavia, 27100 Pavia, Italy; augusto.arrighi@hotmail.com; 5 San Rossore Dental Unit, Viale delle Cascine 152, San Rossore, 56122 Pisa, Italy; 6 Department of Health Sciences, Pegaso Digital University, 00187 Rome, Italy

**Keywords:** diabetic patients, dental implants, glycated hemoglobin, implant rehabilitation, metabolic disorders

## Abstract

Advancements in implant design, surface characteristics, and surgical protocols have made implant restorations safe and highly predictable procedures. Bone metabolism plays a central role in the success of implant therapy. Diabetes mellitus is a significant disease impacting bone metabolism, particularly during the initial stages of osseointegration and in long-term survival. Moreover, aging is linked to various systemic conditions, such as diabetes, which increase the susceptibility of the periodontium and teeth to disease, often resulting in tooth loss. Studies on the impact of glycemic control on the success and longevity of implant–prosthetic rehabilitation in diabetic patients highlight a significant association between hyperglycemia and complications in implant therapy. This review identified 18 relevant publications through Medline, and studies were screened against the aim and objectives of the review. A total of five articles were excluded because of lack of focus on the effect of glycemic control on dental implants. Diabetic patients with poorly controlled blood glucose levels may face a heightened risk of developing implant complications. Effective glycemic control plays a critical role in the long-term success of dental implants in these individuals. Marginal bone loss (MBL) is a critical indicator of implant health and success. Clinical studies generally show greater MBL in diabetic patients compared to non-diabetic individuals. Furthermore, controlled type 2 diabetes mellitus (T2DM) has been associated with significantly better maintenance.

## 1. Introduction

Dental implants represent the gold standard for replacing missing or hopeless teeth that have to be removed [1]. Advances in implant design, surface properties, and surgical protocols have established implant restoration as a safe and highly predictable treatment, achieving an average survival rate of 94.6% and a success rate of 89.7% over a ten-year follow-up period [2].

The survival of dental implants initially depends on achieving primary stability and successful osseointegration after placement. Any disruption to these biological processes can negatively affect treatment outcomes. Once an implant is fitted with a prosthesis and subjected to functional load, bone remodeling becomes crucial for implant longevity, allowing adaptation to the mechanical demands placed on both the implant and the surrounding bone. Proper implant positioning, meticulous surgical planning, and consideration of bone metabolism are essential for the long-term success of implant therapy [2].

A particularly contentious issue in implantology is the impact of diabetes mellitus on bone metabolism. Diabetes may influence osseointegration during the initial stages and contribute to bone loss, affecting the long-term survival of implants [3,4].

Globally, life expectancy has significantly increased. The World Health Organization reports a current global average life expectancy of 72.8 years, compared to 59.7 years five decades ago [5].

Aging has a direct impact on oral health, with adverse effects related to various diseases and medication regimens. Consequently, teeth become more susceptible to disease processes and eventual tooth loss [3,4,5].

Historically, the replacement of a missing posterior tooth most commonly involved a fixed partial denture (FPD); however, FPDs present certain risks, including limited restoration longevity, and, most importantly, the possible failure of the abutment teeth supporting the denture [6,7].

Several studies in the literature indicate that fixed prothesis is a preferred treatment option even in cases of atrophic maxilla compared to removable protheses due to an improvement in patients’ quality of life; nowadays, difficult implant cases for patients with bone atrophic and systematic diseases are more frequent because patients have higher standards for a satisfying lifestyle [8,9,10,11].

Diabetes mellitus is a group of metabolic diseases characterized by hyperglycemia (high levels of blood glucose) which results from different hormonal defects such as insulin secretion (i.e., the pancreas cannot produce enough insulin), insulin effect (i.e., the cells cannot effectively utilize the insulin produced by the body), or both. It is a global public health challenge with high prevalence. The most common type of diabetes mellitus is type 2, which accounts for 90–95% cases. In 2021, it was estimated that 537 million adults worldwide were affected, with projections indicating this will reach to 640 million by 2035 [12,13].

Long-term hyperglycemia is commonly associated with a range of complications that affect multiple organs and tissues, leading to significant clinical morbidity [13,14,15]. The duration of diabetes is linked to the worsening of both oral and systemic health, exacerbating clinical issues even in patients with well-controlled blood glucose levels, and this progression is not always age-dependent [14,15,16,17]. These morbidities are often the result of various negative effects of diabetes, including delayed wound healing, microvascular complications, impaired immune response, and disrupted bone metabolism [14,15,16,17,18].

For individuals diagnosed with type 2 diabetes (T2DM) at a young age, the risk of microvascular and other complications increases over time [19,20]. Changes in microvascular function associated with T2DM appear to negatively affect immune response, compromising the vascularization of tissue flaps. This leads to delayed healing and increases the risk of peri-implant soft tissue infections [20,21].

Blood glucose levels are key in the development of systemic complications related to diabetes. The relationship between glycemic control and the onset of microvascular and macrovascular complications has been extensively studied [21]. Good glycemic control can delay the onset and progression of many microvascular complications. However, the effectiveness of this control in preventing complications diminishes once they have already developed [18,21,22]. The average blood glucose level over the past three months is assessed through hemoglobin A1c (HbA1c) level, a test that measures the percentage of hemoglobin proteins that are glycated. In individuals with diabetes, this value should be maintained below 6.5% to be considered under control [23,24].

Oral complications of diabetes significantly increase the risk of partial or complete tooth loss. These complications are multifactorial, including gingivitis, periodontal disease, xerostomia, increased susceptibility to infection, dental caries, and periapical lesions, all of which contribute to higher rates of tooth extraction according to the guidelines of periodontology published in 2023 [24,25].

In diabetic and prediabetic individuals, the destruction of periodontal and peri-implant tissues is primarily driven by an inflammatory and immune response triggered by oral microbiota dysbiosis. Research suggests that hyperglycemia in diabetes is associated with the excessive production and accumulation of advanced glycation end-products (AGEs), which impair the synthesis of matrix proteins (such as collagen and osteocalcin) by fibroblasts. This results in delayed wound healing and structural damage to periodontal tissues. Additionally, the interaction between AGEs and their receptors activates the expression of inflammatory cytokines, such as IL-1β, TNF-α, and IL-6, in the gingival crevicular fluid and serum. These cytokines exacerbate inflammation and promote bone loss around both teeth and dental implants [18,20,21,22,23,24,25].

Chronic infection increases the risk of worsening metabolic control, which in turn can exacerbate periodontal disease due to oxidative stress. As a result, dental clinicians may anticipate the need for tooth extraction [26]. Tooth loss can impair nutrition by making chewing difficult, affect speech, and negatively alter facial appearance, leading to potential social challenges. Therefore, treatment with dental endosseous implants provides an effective solution, significantly enhancing patients’ perceived quality of life, but the literature is not homogeneous regarding patients affected with diabetes.

In individuals with healthy dentition, the average biting force of the first molars is relatively high. However, for edentulous patients using removable complete dentures, this force can decrease by up to 80%. Among these patients, 29% can only consume soft or mashed foods, 50% avoid certain types of food entirely, and 17% report feeling more comfortable eating without their dentures. In contrast, multiple studies have highlighted the benefits of implant-supported prostheses, particularly in terms of patient satisfaction and perceived quality of life [27,28].

Furthermore, some studies have emphasized that fixed implant prostheses can help preserve bone loss following tooth extraction [29]. Numerous clinical studies and reviews have demonstrated that rehabilitating edentulism with implant-retained or fixed prostheses results in significant improvements in patients’ quality of life; here, patient satisfaction levels are notably higher compared to those rehabilitated with conventional removable dentures [30,31,32], but still there is a lack of information regarding diabetic patients.

The aim of the present comprehensive narrative review is to synthesize global evidence from in vivo and clinical studies as well as reviews evaluating the impact of glycemic control on dental implant treatment.

## 2. Materials and Methods

The electronic database search for this narrative literature review was performed on Medline using the following search terms in different combinations of keywords: “dental implant”, “type 2 diabetes” or “hyperglycemia”, “osseointegration”, or “Peri-implantitis” or “marginal bone loss”, and “glycemic control”. The intent of the authors was to be as comprehensive as possible in a narrative-type review and to be sure that all relevant studies that have been conducted are included. All the common databases have been searched such as MEDLINE (via PubMed), Embase, and the Cochrane Central Register of Controlled Trials. Thus, we included reviews, clinical studies, and in vitro studies on animal models.

The questions addressed by the authors in this narrative review were the following:

1. Should quality of glycemic control guide dental implant treatment in patients affected by diabetes?
2. What is the long-term effect of hyperglycemia on dental implants (i.e., implant survival rate)?

The null hypothesis was that diabetic patients with poor glycemic control would not have higher rate of complications with dental implants.

The primary inclusion criteria focused on studies published within the past 20 years that investigated the impact of glycemic control on outcomes related to dental implant surgery and prosthetic rehabilitation in patients with type 2 diabetes, as follows:

- Implant failure: Implant failure is the inability of an implant to maintain or achieve the intended integration with surrounding tissue, often resulting in clinical symptoms such as pain, mobility, or inflammation. It is categorized as early when osseointegration fails during the initial healing phase; it is categorized as late when the established integration degrades over time due to factors like mechanical stress or infection.
- Osteointegration: The establishment of a direct structural and functional interface between bone and the surface of a load-bearing implant is essential for achieving secondary implant stability. This stability is dependent on the dynamic remodeling of the surrounding bone, which involves osteoblast proliferation and the subsequent formation of new bone tissue.
- Peri-implant diseases: These include inflammation characterized by an inflammatory response within the peri-implant soft tissues, typically evidenced by bleeding on probing, increased probing depths indicative of pathological changes, and progressive marginal bone loss [23].
- Marginal bone loss (MBL): MBL refers to the gradual reduction in the bone volume surrounding the implant, typically observed at the bone–implant interface, which can lead to compromised implant stability and function, often caused by factors such as poor oral hygiene, infection, overload, or peri-implantitis.
- Implant Survival Rate: The number of dental implants that did not fail >5 years or were not removed during the follow up [31,32,33,34,35].
- Glycemic control based on glycated hemoglobin (HbA1c): Well-controlled—HbA1c less than 7.0% (53 mmol/mol); moderately controlled—HbA1c between 7.0% and 8.0% (53–64 mmol/mol); poorly controlled—HbA1c greater than 8.0% (64 mmol/mol).

Articles published in languages other than English and any grey literature studies were excluded due to limited accessibility and insufficient resources for synthesis.

## 3. Results

The literature search through Ovid Medline identified 18 articles. Studies were screened according to the inclusion and exclusion criteria and a total of five studies were excluded at full-text screening stages because of their lack of focus on the primary endpoint, i.e., the effect of glycemic control on dental implants.

Among the included articles, ten were review studies, and eight were experimental studies (comprising both clinical and animal trials). The results of these studies are not directly comparable due to the differing nature of the papers; however, their conclusions are reported as follows [3,23,36,37,38,39,40,41,42,43,44,45,46,47,48].

In a 2021 systematic review, Aldahlawi S et al. reported inconsistent findings concerning the long-term impact of diabetes on peri-implant tissues in relation to glycemic control levels. Consequently, dental treatment planning for diabetic patients should be personalized to account for these variable outcomes and individual patient needs [36]. Nourah D and collaborators, in a recent systematic review (2022), also stated that evidence for the survival or success of implants in diabetic patients is inconsistent due to a lack of standardized clinical data collection and outcomes [37]. Some studies reported that implant treatments in patients with well-controlled diabetes have similar success and survival rates to healthy individuals, while other studies suggested that the quality of glycemic control in diabetic patients does not make a difference in the implant failure rate or marginal bone loss [37].

In a recent systematic review (2022), Andrade CAS and colleagues suggested that Type 2 diabetes mellitus may not pose a risk factor for immediately loaded implants when glycemic levels are well-controlled, and oral hygiene is maintained at a high standard. Under these conditions, implant survival rates were reported to range from 86.3% to 100% [38].

A study of Li H and colleagues in 2021 evaluated different levels of glycemic control to determine whether glycemic fluctuation has greater adverse effect on experimental peri-implantitis. This study suggests that glycemic fluctuations may exacerbate peri-implantitis-related inflammation and bone loss, potentially due to a shift in the peri-implant microbial profile toward dysbiosis [39].

The 2021 systematic review and meta-analysis by Tan SJ et al. highlighted moderate evidence of a possible dose–response trend indicating a decline in implant clinical and radiological outcomes with increasing HbA1c levels. The study reported implant survival rates between 92.6% and 100% and success rates ranging from 86.3% to 95.4%.

Lagunov VL (2019) and others have reported that Type 2 diabetes mellitus (T2DM) may present a significant risk factor for dental implants, even in cases where glycemic levels are controlled within an HbA1c range of 6.1% to 8%. Conversely, other studies have indicated that dental implants can achieve successful osseointegration and maintain long-term stability in patients with diabetes [40,41,42].

Other studies have demonstrated significantly greater hard and soft tissue loss in patients with elevated glycemic levels. Patients with poorly controlled diabetes exhibit a higher risk of peri-implantitis and implant failure [43,44,45,46,47,48].

### 3.1. Diabetes Mellitus and Osseointegration

Most studies included in the review suggest that osseointegration is slower in diabetic patients with poor glycemic control. These patients typically require a longer healing period, often at least six months, before implant loading [35,36,37,38,39,40]. Persistent hyperglycemia plays a significant role in the abnormal differentiation of osteoclasts, thereby increasing the risk of bone tissue resorption. If hyperglycemia remains untreated, it may compromise implant stability. Specifically, if the bone healing and osseointegration processes occur too slowly in comparison to the loss of primary stability, this can lead to implant failure due to the inability to achieve secondary stability [42,47,48,49,50,51,52,53,54].

The majority of the literature suggests that osseointegration can occur successfully with optimal glycemic control. However, in cases of uncontrolled diabetes, animal studies have shown that bone-to-implant contact (BIC) is less effective, as indicated by the reduced expression of integrin α5β1 and fibronectin in the bone surrounding the implant [45,55,56]. A recent systematic review of clinical studies examining implant stability one year after implant surgery found no significant differences between various glycemic levels, including poorly controlled HbA1c. While implant stability measurements were heterogeneous, resonance frequency analysis was the most commonly used method [4,57,58,59]. Additionally, factors such as the use of antiseptic mouth rinses and effective oral hygiene maintenance play a crucial role in the successful osseointegration of dental implants in diabetic patients [26,40,41,42,43,44,45,46,47,48].

### 3.2. Glycemic Control and Peri-Implant Diseases

Compared to the general population, diabetic patients exhibit altered host responses and environmental factors that contribute to a shift in the peri-implant microbial profile, promoting dysbiotic changes that facilitate excessive bacterial plaque accumulation. This, in turn, leads to a more pronounced progression of peri-implant disease [36]. Additionally, the degree of glycemic control has been positively associated with the development of peri-implantitis [35,36,37,39,40]. However, other studies have contradicted this association, suggesting no significant link between diabetes and peri-implant disease [4,38].

### 3.3. Diabetes Mellitus and Marginal Bone Loss

Marginal bone loss (MBL) is widely regarded as a key indicator for evaluating the health and success of dental implants. Clinical studies have generally shown a significant difference in MBL between patients with and without diabetes. Additionally, some studies have reported that patients with controlled Type 2 diabetes mellitus (T2DM) exhibit significantly higher MBL compared to non-diabetic individuals. When assessing MBL, factors such as the duration of follow-up and the specific procedures or techniques employed should also be taken into account [38,41].

## 4. Discussion

According to the International Diabetes Federation, DM has been defined as “a long-term condition that occurs when raised levels of blood glucose occur because the body cannot produce any or enough of the hormone insulin or cannot effectively use the insulin it produces” [15].

Compared to healthy individuals, hyperglycemia poses a risk on the survival rate of dental implants [4,42,43,44,45,46]. This is because patients with poorly controlled diabetes suffer from impaired osseointegration, elevated risk of peri-implantitis, and higher level of implant failure [47]. When glycemia is well controlled, dental implants are safe, and dental implant survival rate and complications are better compared to those of non-diabetic patients. However, the effect of glycemic control on dental implants documented in the literature is inconsistent. Some studies strongly suggest that glycemic control determines the survival rate of dental implants and must be considered during follow-up in a patient with diabetes [40]. A study that considered the level of glycemic control among type 2 diabetic patients reported that well-controlled glycemia had a 100% survival rate when other risk factors are controlled, while survival rate in poorly controlled diabetics dropped to 86.3% (32, 38, 44). Therefore, this finding must be examined carefully [36,37]. Most authors agreed that proper domiciliary oral hygiene and short-term follow-up reduce the risk of a higher level of inflammation; accordingly, this reduces the risk of hyperglycemia. It is important to give clinicians the right to conduct check-ups for implant–prosthetic rehabilitation [26,40,41,42,43,44,45,46,47,48,49,50].

Studies have consistently shown that patients with poorly controlled diabetes exhibit a higher prevalence of peri-implant diseases, such as peri-implant mucositis and peri-implantitis. These inflammatory conditions are more severe in diabetic patients due to the enhanced inflammatory response associated with hyperglycemia. Abduljabbar et al. (2017) compared peri-implant inflammatory parameters among prediabetic, diabetic, and non-diabetic individuals and found significantly higher levels of inflammation in diabetic patients, particularly those with poor glycemic control [16].

The impact of glycemic control on the success of dental implants is further supported by the findings of Aldahlawi et al. (2021), who emphasized that the quality of glycemic control should be a guiding factor in implant therapy [36]. Their study found that diabetic patients with better-controlled blood sugar levels (as measured by HbA1c) experienced fewer complications and better overall implant outcomes. This suggests that achieving and maintaining optimal glycemic control before and after implant placement is crucial for minimizing the risk of peri-implant diseases and ensuring the long-term success of the implants [37].

The process of osseointegration, where the implant integrates with the surrounding bone, is critical for the stability and success of dental implants. Hyperglycemia negatively impacts this process by inhibiting bone formation and promoting bone resorption. Mellado-Valero et al. (2007) demonstrated that hyperglycemia impairs the osseointegration of dental implants, leading to a higher risk of early implant failure [35]. This is particularly concerning given that bone loss around implants is a major predictor of long-term implant failure.

In diabetic patients, the risk of peri-implant bone loss is exacerbated by poor glycemic control. Ghiraldini et al. (2016) found that diabetic patients with uncontrolled blood sugar levels experienced significantly greater bone loss around their implants compared to patients with well-controlled diabetes and non-diabetics [44]. This bone loss not only compromises the stability of the implant but also increases the likelihood of implant failure over time. These findings underscore the importance of glycemic control in managing bone health and ensuring the long-term success of dental implants in diabetic patients.

Chronic hyperglycemia has far-reaching effects on the success and longevity of dental implants. The long-term survival rate of implants in diabetic patients is significantly lower than that in non-diabetic individuals, primarily due to the detrimental effects of high blood glucose levels on bone metabolism and immune response.

Numerous studies have explored the relationship between hyperglycemia and implant survival rates. Dubey et al. (2013) conducted a comprehensive review of dental implant survival in diabetic patients and found that those with poorly controlled diabetes had significantly lower implant survival rates compared to non-diabetic controls [18,19,20,21]. The authors attributed this to the negative effects of chronic hyperglycemia on bone healing and immune function, which are critical for implant integration and long-term stability.

Chambrone and Palma (2019) provided further evidence of the impact of diabetes on implant survival, noting that uncontrolled T2DM is associated with higher rates of peri-implant bone loss and implant failure [20]. Their findings are supported by a meta-analysis conducted by Andrade et al. (2022), which showed that diabetic patients with well-controlled glycemic levels had implant survival rates comparable to those of non-diabetic individuals [38]. However, the survival rate dropped significantly in patients with poor glycemic control, highlighting the importance of maintaining stable blood glucose levels to ensure the long-term success of dental implants.

The chronic inflammatory state induced by hyperglycemia plays a key role in the increased incidence of implant failure in diabetic patients. Li et al. (2021) studied the effects of glycemic fluctuations on inflammation and bone loss around implants in diabetic mice, demonstrating that hyperglycemia exacerbates both local inflammation and bone resorption [39]. This inflammatory environment is detrimental to implant stability, as it promotes the breakdown of the peri-implant bone, leading to a higher likelihood of implant failure.

Moreover, Javed and Romanos (2019) highlighted the role of advanced glycation end products (AGEs) in the pathophysiology of diabetes-related complications, including those affecting dental implants. AGEs accumulate in tissues as a result of chronic hyperglycemia and contribute to the degradation of collagen and other structural proteins in bone and soft tissues [42,46]. This degradation weakens the bone–implant interface and impairs the osseointegration process, further increasing the risk of implant failure.

Given the evidence presented, it is clear that the quality of glycemic control is a critical determinant of the success and longevity of dental implants in diabetic patients. Clinicians should prioritize the management of blood glucose levels before, during, and after implant placement to minimize the risk of complications and enhance implant survival rates.

Preoperative assessment of glycemic control should be a standard practice in the treatment planning of dental implants for diabetic patients. This includes regular monitoring of HbA1c levels and other markers of glycemic control. Patients with poorly controlled diabetes should be advised to postpone implant surgery until their glycemic levels are stabilized, as this will reduce the risk of peri-implant complications and improve the likelihood of successful osseointegration.

In addition to glycemic control, other factors such as smoking cessation, management of comorbidities, and optimization of overall oral health should be addressed preoperatively. Sanz et al. (2018) recommend a multidisciplinary approach involving endocrinologists, dentists, and periodontists to ensure that all aspects of the patient’s health are considered in the treatment plan [15].

Postoperative care is equally important in ensuring the long-term success of dental implants in diabetic patients. Regular follow-up appointments should be scheduled to monitor the implant site for signs of inflammation, bone loss, or other complications. Patients should be educated on the importance of maintaining good oral hygiene and glycemic control to prevent peri-implant diseases.

The use of adjunctive therapies, such as antimicrobial agents and anti-inflammatory medications, may also be beneficial in managing peri-implant health in diabetic patients. Periodontal interventions and implant rehabilitation after removing infected teeth could potentially enhance the long-term success of dental implants in this population [26,58].

The present review has shown a potential increased risk of peri-implant diseases in diabetic patients, but a limitation of the present study is that we did not distinguish between several types of bone density and quality in either the upper or lower jaw, or the anterior or posterior site. Obviously, the prevalence of peri-implantitis in the literature has been reported on in such a way that notes differences among age and gender, type of implant prosthesis connection, region due to the different function, available native bone, and different oral hygiene statuses; but, even though the main focus of this review was related to the quality of glycemic control in diabetes, this is an important factor for the success of dental implants both in terms of mean glycemia and high-level glycemia after stimulus [24,56,57,58,59]. In experimental models of diabetes, a reduced level of bone–implant contact has been shown, which was reversed by means of treatment with insulin. On the other hand, poorly controlled diabetes has poorer outcomes with dental implants compared with the non-diabetic population. Clinical and radiologic markers of peri-implantitis, the plaque index, probing depth, bleeding on probing, marginal bone loss, and salivary interleukin levels were increased in diabetic patients [20]. Hence, higher glycemic levels (HbA1c > 8%) were found to be associated with greater prevalence of compromised stability of implants and worse healing of soft tissue [38]. Therefore, in cases of diabetic patients, it is necessary to consider and to reduce other risk factors, especially in the first year of functional loading, when most microvascular complications which compromise the healing of soft tissues occur [20]. Moreover, controlling other associated factors has been shown to improve the percentages of implant survival in these patients [35].

Concerning the long-term impact of diabetes and the level of glycemic control, our review identified studies that reported on the negative effect of poorly controlled glycemia on the survival rate of dental implants. Supporting our findings, another study stated that poorly controlled type 2 diabetes seems to negatively influence the survival rate of dental implants [51]. In a study that evaluated the association between well-controlled diabetes and implant survival rate, there was no significant correlation [52].

Another limitation of the present study is related to the nature of the study; it was not designed as a systematic review because the scope was to be as inclusive as possible in terms of clinical and in vivo studies; however, this means we have compromised in terms of obtaining comparable results. The next potential step to overcome this limitation will be to perform an updated systematic review with a meta-analysis.

However, the effect of glycemic control on dental implants documented in the literature is heterogenous; further clinical studies are needed because there are too many variables related to the patients, the implants, and the surgeons [53,54]. This recommendation was supported by other primary studies that reported heterogeneous results of the influence of glycemic control on peri-implantitis; here, peri-implantitis was found to be pronounced in poorly controlled glycemia, yet these complications were not significant. Accordingly, retrospective studies are required to ensure a better understanding of the epidemiology of implant failures, which should be adjusted by the type of surgery, the type of implants, and the type of protheses [55,56].

Despite what the literature reported, the common situation is that diabetic patients are followed-up with by a multidisciplinary team, including general practitioners, endocrinologists, diabetologists, dietitians, podiatrists, and physiotherapists; dentists are not often included as part of this care team, despite the impact of poor oral health on glycemia control [57,58,59,60]. Considering the results of this review, both diabetes professionals and dental professionals have an excellent opportunity to collaborate and to raise awareness among patients with diabetes about their increased risk of oral health diseases and motivate them to have good oral hygiene behaviors and regular dental check-ups and treatments, even dental implants. Healthcare professionals working with patients with diabetes should play a more active role in promoting oral health among their patients, showing the chance to perform implant–prosthetic rehabilitation in cases of diabetes, and reporting on the importance of oral hygiene and maintenance. Professionals should educate patients about their increased risk of oral health complications and advise them to have regular dental check-ups.

Oral health professionals should educate people with diabetes to have good oral health behavior, emphasizing the importance of having regular dental check-ups, removing local infections, and ensuring good glycemic control, minimizing the risks for oral health.

## 5. Conclusions

- Marginal bone loss is worse in patients with uncontrolled diabetes compared to non-diabetic individuals.
- However, the radiological and clinical outcomes of dental implant placements in well-controlled type 2 diabetes patients are encouraging.
- The current literature suggests no significant differences in implant success between well-controlled diabetic patients and non-diabetic individuals.
- Optimizing glycemic control and addressing other risk factors can improve implant survival rates and overall outcomes in diabetic patients.
- A multidisciplinary approach is essential for managing diabetic patients undergoing dental implant therapy, involving various healthcare professionals.
- Further well-controlled long-term clinical trials and retrospective studies are needed to achieve the following goals: compare patients with different glycemic profiles and fluctuations in blood glucose levels; use consistent dental and medical indicators to optimize implant surgery and prosthetic maintenance strategies.

## Data Availability

No new data were created.
